# Syringohydromyelia in Dogs: The Genomic Component Underlying a Complex Neurological Disease

**DOI:** 10.3390/ani12192622

**Published:** 2022-09-29

**Authors:** Sandra Andrino, Valentina Lorenzo, Susana Dunner, Elisabeth Contreras, Javier Cañón, Natalia Sevane

**Affiliations:** 1 Neurología Veterinaria, C. Del Diseño 26, Nave 39, Getafe, 28906 Madrid, Spain; 2 Departamento de Producción Animal, Facultad de Veterinaria, Universidad Complutense de Madrid, Av. Puerta de Hierro s/n, 28040 Madrid, Spain

**Keywords:** canine, genome-wide association study (GWAS), hydrosyringomyelia, spinal cord, syringomyelia

## Abstract

**Simple Summary:**

Syringohydromyelia is a neurological disorder which is characterized by the appearance of fluid-containing cavities within the spinal cord and is common in brachycephalic dogs. Although syringohydromyelia is believed to be under multigene control, the molecular basis of this disease remains poorly defined. In this study, seven candidate genes were identified. Of these, five genes were determined to be involved in bone development (*PLXNA2*, *HHAT*, *MBOAT2*, *ITGAV*) and calcium homeostasis (*HPCAL1*). Considering previous studies in dogs showing syringohydromyelia associated with craniocervical junction malformations, these genes may be considered good candidates for the development of this disease. This report dissects the genomic component of syringohydromyelia in dogs, paving the way for further research into this complex disease found in both canine and human species.

**Abstract:**

Syringohydromyelia (SHM) is a neurological disorder characterized by the appearance of fluid-containing cavities within the spinal cord. Although SHM is thought to be under multigenic control, the molecular basis of this disease remains poorly defined. A genome-wide association study (GWAS) was carried out comparing the whole genome sequences (WGS) from 12 dogs with SHM and 2 panels of 26 dogs (either older than 5 years and showing the absence of SHM or belonging to breeds not susceptible to SHM) to identify candidate genes associated with the development of SHM. Seven candidate genes were identified. Of these, five genes were determined to be involved in bone development (*PLXNA2*, *HHAT*, *MBOAT2*, *ITGAV*) and calcium homeostasis (*HPCAL1*). Although further validation is needed at the transcript level, it is worth highlighting the association of a possible pathogenic variant which generated a new intronic branch-site sequence in *PLXNA2* (T/C, CFA7:7043294). Considering previous studies in dogs that show SHM related to craniocervical junction (CCJ) malformations, these genes can be considered good candidates for the development of this disease. This report dissects the genomic component of SHM in dogs, which paves the way for further research on this complex disease found both in canine and human species.

## 1. Introduction

Syringomyelia (SM) is a progressive neurological disorder characterized by the appearance of fluid-containing cavities (syringes) within the parenchyma of the spinal cord (SC) caused by abnormal flow of cerebrospinal fluid (CSF) [1]. The abnormal dilatation of the central canal, with an intact ependymal layer, is referred to as hydromyelia (HM) and is regarded as the preliminary stage of SM. Given that differentiation between the two entities is anatomical, the terms hydrosyringomyelia (HSM) and syringohydromyelia (SHM) have been used historically in the clinical field. However, the term syringomyelia has been commonly used for both conditions in last years [2]. For the purpose of this study, the term SHM is considered.

This condition is common in brachycephalic toy dog breeds such as Cavalier King Charles Spaniels (CKCS) (with a 25% prevalence in dogs aged 12 months, increasing to 70% in dogs aged 72 months or older) [3], Griffon Bruxellois (GB) (8.9–37.5%) [4], or Chihuahuas (38%) [5], and is often related to craniocervical junction (CCJ) abnormalities, such as Chiari-like malformation (CM), atlanto-occipital overlapping, atlanto-axial instability, and SC dorsal compression [6]. In humans, the vast majority of cases of SHM are associated with an abnormality at the foramen magnum as well (particularly the Chiari malformation type I) [7]. However, it has also been related to other problems such as achondrodysplasia [8]. Other known risk factors, described in human medicine and also present in dogs, are spinal canal stenosis and kyphosis [9].

Depending on the width and location of the SHM, the clinical signs may include scratching at the neck/shoulders, spontaneous vocalization, scoliosis, thoracic and/or pelvic limb ataxia and weakness, and urinary/fecal incontinence, among others [1]. The disease shows variable degrees, with the most severe cases presenting substantial SC damage and significant disability. Conversely, others dogs show an asymptomatic SHM [3]. The severity is often associated with large syringes (e.g., in CKCS a maximum transverse width equal to or greater than 4 mm). However, gait disturbance may be mild even in cases involving the entire spinal canal [10].

Although CCJ abnormalities are considered to play a major role in its development, SHM is often thought to be a multifactorial disease under a multigenic control with moderately to high heritability, as shown by studies in some breeds such as the CKCS [11]. However, the molecular basis of this disease remains poorly defined. Some genome-wide association studies (GWAS) aiming at identifying genes or SNPs related to the development of SHM with CM have been conducted, identifying, on the one hand, two quantitative trait loci (QTLs) on CFA2 (associated to the cranial fossa height; *Sall-1* gene) and CFA14 (associated to the rostral of the caudal cranial fossa height and brain height) in GB [12]. On the other hand, two QTLs were identified on CFA22 (*PCDH17*) and CFA26 (*ZWINT*) in CKCS [13]. Thus, this study aims to identify genomic regions and candidate genes predisposing to SHM.

The French Bulldog (FB), a brachycephalic and chondrodystrophic breed, has a high prevalence of neurological diseases, such as intervertebral disc disease (IVDD), vertebral malformations (that may induce canal stenosis and kyphosis), and spinal arachnoid diverticulum [14,15], which may be associated with SHM. In this breed, Ricco et al. [16] reported a prevalence of 67% for CM, 42% for spinal dorsal compression at C1-C2, 42% for cervical disc herniation, and 8% for SHM. In addition, FBs share morphological features of the skull with breeds with a high predisposition for SHM such as CKCS or GB. In fact, this breed gathers 129 out of 330 SHM cases (39%) attended between 2011 and 2019 in the Veterinary Neurology Reference Center (VNRF) where the study was carried out. For the purpose of the study, two GWAS were conducted in dogs by comparing 12 FBs with SHM with 2 different panels of 26 controls each (10 FB older than 5 years and showing the absence of SHM plus 16 dogs from control panel 1 (CP1, n = 16, breeds = 9) or control panel 2 (CP2, n = 16, breeds = 5) belonging to breeds not susceptible to SHM). Different candidate genes arose, which may have relevant effects in the onset and development of this disease.

## 2. Materials and Methods

### 2.1. Animals and Sample Collection

Approval by an ethics committee for the use of the blood samples was not necessary. All animals were examined with the signed consent of their owners in a VNRC from February 2019 to March 2021. Dogs in the SHM group were 12 FBs of any age diagnosed with SHM (cervical, thoracic or/and lumbar) by magnetic resonance imaging (MRI), regardless of the presence of other spinal diseases (Table 1). Controls were 10 FBs (Table 1) plus 32 dogs belonging to other established breeds and divided in two small panels of 16 samples to perform two GWAS (Table S1). These additional controls from different breeds were used to increase the detection power of GWAS, as suggested by other authors [17]. The samples included in each control panel were different (Table S1): control panel 1 (CP1) included WGS data from 16 dogs belonging to nine established breeds (3 Sloughi, 2 Dalmatian, 1 German Wirehaired Pointer, 2 Gordon Setter, 1 Great Pyrenees, 1 Komondor, 2 Shiba Inu, 3 Siberian Husky, 1 Weimaraner); and control panel 2 (CP2), 16 individuals from five different breeds (3 Bouvier des Flandres, 3 English Setter, 4 Great Dane, 4 Rottweiler, 2 Saint Bernard). The 10 FBs controls were 5 years of age or older (based on mean ages from other neurological studies in FBs; [14]), with MRI of the complete spine showing the absence of SHM regardless of the presence of other spinal diseases (Table 1). The 32 additional control samples were extracted from the VCF file with NCBI accession number PRJNA448733 [18] and no MRI data is available for them. The selection criteria to include this samples as controls were as follows: (1) breeds with a low prevalence of SHM described in the literature and the VNRC database; (2) breeds that were least related and that shared few or no haplotypes with those breeds that had cases of SHM (based on the cladogram and the haplotype sharing between phylogenetic clades of dog breeds from Parker et al. [19]); (3) ≥10X coverage; and (4) the two males and two females that had the deepest coverage (when more than three individuals by breed were available). 

All dogs from the VNRC were diagnosed by a certified neurology specialist based on clinical signs and imaging, and classified according to the neurological dysfunction grade (Table 1). MRI of the spine was performed with a 1.5 T unit (Gyroscan Intera, Philips, Eindhoven, The Netherlands). Images included at least sagittal and transverse T2-WI sequences and T1-WI. Information on age, sex, body weight and definitive diagnosis were collected (Table S1). Blood samples were collected and preserved in Magic Buffer^®^ (Biogen Diagnostica, Madrid, Spain) at room temperature. Genomic DNA was isolated using the E.Z.N.A.^®^ Blood DNA Mini Kit (Omega Bio-Tek, Norcross, GA, USA) according to the manufacturer’s instructions. DNA concentration was measured with Implen NanoPhotometer™ (BioNova, Madrid, Spain). The samples were kept at −20 °C until use.

### 2.2. Whole-Genome Resequencing

WGS data were generated from 12 FB SHM cases and 10 FB controls collected in the VNRC using the BGISEQ-500 platform with an average sequence coverage of 10.5X and following a 150 pb pair-end protocol. After sequencing, the raw reads were filtered, removing adaptor sequences, contamination and low-quality reads from raw reads using the SOAPnuke software developed by BGI, deleting the entire reads if more than 25% match the adapter sequence, more than 50% bases have a quality value lower than 20, or there are more than 3% N in the read. Clean data is available on NCBI accession number PRJNA825150 and statistics are shown in Table S2. Clean sequence reads were aligned to the canFam5 (UMICH_Zoey_3.1) reference genome using the BWA-MEM algorithm [20] (version 0.7.17) and sorted with SAMtools [21] (version 1.14). PCR duplicates were marked as secondary reads using PicardTools (http://github.com/broadinstitute/picard (accessed on 22 September 2022); version 2.26.1) (Table S3). Freebayes [22] (version 1.3.5) was used for haplotype-based variant detection with the following parameters: --gvcf -g 500 -C 5. Variants with estimated probability of not being polymorphic less than phred 20 and coverage deep below 10 were removed using vcffilter in vcflib [23] (version 1.0.2). As variants for samples from Plassais et al. [18] were called on the CanFam 3.1 reference genome, we used the LiftoverVcf Picard tool to adjust the coordinates of variants within our 22 samples’ VCF file to match this genome build, and bcftools [24] (version 1.14) to merge both datasets. Only variants with a minor allele frequency (MAF) above 5% and a call rate of at least 99% across all samples were retained for downstream analyses using PLINK 2.0 [25] (http://github.com/broadinstitute/picard, www.cog-genomics.org/plink/2.0/ (accessed on 26 October 2021)). 

### 2.3. Genome Wide-Association Studies (GWAS)

We performed 2 GWAS analyses: (a) 12 SHM cases vs. 26 controls (10 simultaneously sequenced with SHM cases within this study plus the 16 included in CP1); (b) 12 SHM cases vs. 26 controls (10 plus 16 from CP2) (Table S1). We used GEMMA [26] (version 0.98.5) as linear-mixed model methods, correcting each analysis by a relatedness matrix previously calculated, the Wald test to determine *P* values and Bonferroni correction to identify significant associations (cutoff = −log_10_ (0.05/number of variants)). By including controls from 14 additional breeds distributed in two GWAS, only the candidate regions showing significant variants in both analyses were considered as significantly associated with the development of canine SHM, thus minimizing the association signals of differences other than susceptibility to SHM. Manhattan plots were constructed in R (http://cran.r-project.org/web/packages/qqman (accessed on 11 November 2021)).

The effect of the intronic candidate variants on a possible exonic splicing enhancer (ESE) was predicted using the web-based tool ESEfinder [27,28] (version 3.0).

## 3. Results

**Figure 1:**
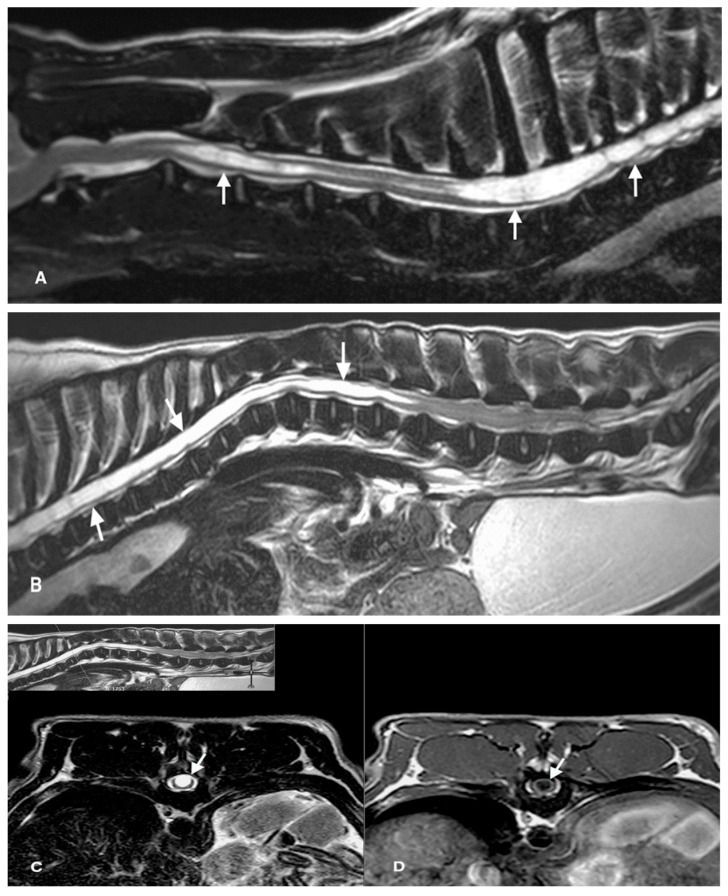
Magnetic resonance imaging (MRI) of the spine of a French Bulldog (FB). An extensive intramedullary cavitary lesion with variable diameter (arrows) is present along the cervical (**A**), thoracic, and lumbar (**B**) segments. The lesion is hyperintense on T2-weighted sequences (**A**–**C**) and hypointense on T1-weighted sequence (**D**). This pattern is characteristic of syringohydromyelia (SHM).

Among the 12 FBs included in this study, SHM was most frequently located in the cervical region (6/12) and in 4/12 of cases the entire SC was affected (Figure 1). The most common clinical signs were gait disturbance with variable degrees of weakness (from ambulatory paraparesis/tetraparesis to non-ambulatory paraparesis/tetraparesis); one case was asymptomatic (Table 1). Both FB cases and controls had other concomitant neurological problems. Of the total number of FB, concomitant spinal diseases included IVDD (20/22), and malformations (15/22 with 8/15 affected and 7/15 unaffected by SHM). The most frequent malformations were thoracic hemivertebrae (15/15), followed by meningocele (2/15). Two dogs affected with SHM had tumors in the brain (no biopsy performed) and in C5 (with inconclusive biopsy), respectively. A detailed description of clinical data is provided in Table 1.

**Figure 2:**
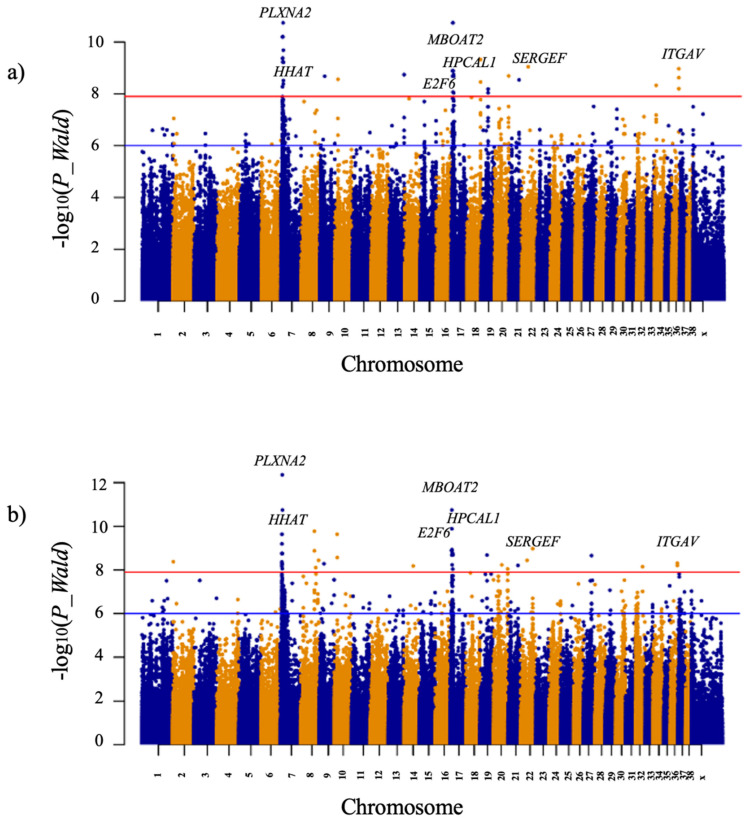
Manhattan plots of the genome-wide association studies (GWAS) for syringohydromyelia (SHM) development comparing (**a**) 12 French Bulldog (FB) SHM cases vs. 26 controls (10 FB, 16 samples from control panel 1), and (**b**) 12 French Bulldog SHM cases vs. 26 controls (10 FB, 16 samples from control panel 2) (Table 1 and Table S1). The −log_10_ *p* values for each SNP are plotted on the y-axis versus each canine autosome and the X-chromosome on the x-axis. The red line represents the Bonferroni corrected significant threshold (−log_10_(P_WALD_) = 7.9) and the blue line the suggestive threshold (−log_10_(P_WALD_) = 6).

GWAS was applied to identify significant variants associated with the risk of developing SHM in dogs using a genome-wide mixed model association algorithm (GEMMA; [26]), which fits a univariate linear mixed model for marker association tests with a single phenotype, using a relatedness matrix to correct for population stratification. We performed two GWAS analyses: (a) 12 FB SHM cases vs. 26 controls (10 FB, CP1: 3 Sloughi, 2 Dalmatian, 1 German Wirehaired Pointer, 2 Gordon Setter, 1 Great Pyrenees, 1 Komondor, 2 Shiba Inu, 3 Siberian Husky, 1 Weimaraner); (b) 12 FB SHM cases vs. 26 controls (10 FB, CP2: 3 Bouvier des Flandres, 3 English Setter, 4 Great Dane, 4 Rottweiler, 2 Saint Bernard) (Table 1 and Table S1). Keeping only variants with minor allele frequency above 5% and a call rate above 99% across all samples, genome-wide data from 3.8 million variants were analyzed. Bonferroni corrections were applied to identify significant associations with a cutoff threshold of 7.9. Only candidate regions displaying significant variants in both analyses were considered as significantly associated with the development of canine SHM, leaving a total of seven candidate genes, three of them located on CFA17 (Table 2) (Figure 2; QQ plots are shown in Figure S1). 

The effect of intron candidate variants on gene splicing was investigated with the ESEfinder webtool, which predicted: a new branch-site sequence not present in the wild type DNA (**T**GGTAAG → **C**GGTAAG, matrix score = 0.86, threshold = 0) for the *PLXNA2* variant (T/C, CFA7:7043294, Canfam 3.1 assembly); and the disruption of a branch-site sequence for the *SERGEF* variant (C/A, CFA21:40329686, Canfam 3.1 assembly, AAT**C**GAC → AAT**A**GAC, matrix score = 1.76, threshold = 0). However, due to the unavailability of the patients’ tissue, we were unable to verify it at transcript level.

## 4. Discussion

SHM is considered a complex disease and is thought to be under multigenic control due to the lack of clear Mendelian segregation within dog families [11], something also confirmed by the outcomes observed in FBs. To avoid possible difficulties in determining nonbiased association results due to the complex and variable status of SHM found in the sample analyzed, we applied 2 GWAS comparing 12 SHM cases with two control groups composed by 10 FBs over 5 years without SHM and 2 panels of 16 dogs belonging to different breeds not susceptible to SHM. The results show that most of the associated genes are involved in bone development. 

Most previous association studies in dogs relate the development of SHM to morphological measures of the skull [12,13]. The results of these studies show association of brachycephaly with the *RUNX2* gene on CFA12 [29], *SMOC2* on CFA1 [30] and *BMP3* on CFA32 [31], chondrodysplasia with *FGF4* retrogene on CFA12 and CFA18 [32,33] and also with skull size [30]. Finally, a single base deletion in the *DVL2* gene has been related to twisted and truncated tail (screw tail), vertebral malformations and brachycephalic phenotype in Bulldogs, FBs and Boston terriers [34,35]. The vast majority of the FBs included in our study, both cases and controls, have other concomitant neurological and morphological problems, the most frequent being IVDD and vertebral malformations, which is in accordance with a high prevalence of these alterations in this breed [14]. Despite the fact that they occur in both groups of dogs, it is known that these traits may collaborate in the development of SHM, but are seldom the direct cause of this condition, as proved by the presence of SHM without other concomitant neurological diseases and vice versa. Therefore, there may be several factors involved in its onset and/or progression, which suggests a complex genetic architecture.

Interestingly, five out of the seven candidate genes identified in this study have been previously involved in bone development (*PLXNA2*, *HHAT*, *MBOAT2*, *ITGAV*) or calcium homeostasis (*HPCAL1*). *PLXNA2* encodes a member of the plexin A family of semaphoring co-receptors (*SEMA3A*, *SEMA6A*). *SEMA3A* is expressed by osteoblasts, where it exerts an osteoprotective effect by suppressing osteoclastic bone resorption and promoting osteoblastic bone formation. Plexin A2 is also expressed in osteoblasts, and it is suggested that sema3A and plexin A2 binding stimulates osteoblast differentiation [36]. Also, as a receptor for sema6A (involved in myelination) [37] and sema3A, *PLXNA2* plays a role in limiting adult axon growth and recovery after trauma [38]. Moreover, it has been shown to have a pro-osteogenic function by modulating *BMP2* signaling via regulation of *RUNX2* [39]. A missense mutation in *BMP3* has been identified as a major contributor to brachycephaly [31], and a repeat expansion of the *RUNX2* gene has been correlated with snout dorsiflexion and midface length in dogs [29]. Recently, Kajii et al. [40] suggested that *PLXNA2* may be a candidate gene for mandibular prognathism since its mutation can delay the early termination of condylar growth. According to this, association of *PLXNA2* with SHM could be explained by affecting CCJ and/or vertebral column bone formation, disturbing CSF flow and consequently inducing the formation of syringes. In our study, the best candidate variant within *PLXNA2* generates a new intronic branch-site sequence (T/C, CFA7:7043294, Canfam 3.1 assembly), which may have a pathogenic effect on the protein, including abnormal exon skipping, retention of the entire intron or its fragment due to the activation of cryptic 3′ splice sites [41]. However, further research at transcript level is needed to validate its possible effect on splicing.

*HHAT* and *MBOAT2* are members of the membrane-bound O-acyltransferase (*MBOAT*) family. Nivelon-Nivelon-Mabille syndrome, also known as chondrodysplasia-pseudohermaphroditism syndrome (OMIM: 600092) is caused by a mutation in *HHAT*. This syndrome presents, among other symptoms, some clinical features shared with FBs, such as dwarfism, chondrodysplasia and trapezoid-shaped vertebral bodies [42]. Furthermore, Dennis et al. [43] demonstrated that loss of *HHAT* function is associated with holoprosencephaly along with acrania and agnatia in mouse models. The enzyme encoded by *HHAT* acts within the secretory pathway to catalyze amino-terminal palmitoylation of ‘hedgehog’. Among Hh family members, loss of *sonic hedgehog* (*SHH*) function results in pathologies as diverse as holoprosencephaly [44] or preaxial polydactyly [45], whereas mutations in *indian hedgehog* (*IHH*) lead to bone growth defects (brachydactyly or acrocapitofemoral dysplasia) [46]. Moreover, a positive feedback loop involving *SHH*, gremlin (a *BMP* antagonist) and *FGF4* is implicated in vertebrate limb growth [47], and some morphogenic molecules such as *SHH*, *BMP*, and *WNT* have a role in SC embryogenesis and their expression is maintained in adults [48]. The other member of the *MBOAT* family, *MBOAT2*, involved in chondrocyte regulation [49], may also influence bone development of dogs with SHM. On the other hand, this gene is implicated in the metabolism of glycerolipids and glycerophospholipids, and has been associated with demyelination [50] and the risk of multiple sclerosis (MS) [51]. 

The last gene implicated in bone development, *ITGAV,* encodes the integrin subunit alpha V, a protein that is involved in osteoporosis, among other processes [52]. The interaction of *ITGAV* with *ITGB3* plays a role in *FGF1* and *FGF2* signaling [53]. *FGF1* and *FGF2*, among other functions, contribute to growth plate chondrogenesis, helping to determine the size of the adult skeleton [54]. Also, a truncated mutation in the cartilage-specific *ITGA10* is known to cause chondrodysplasia in dogs [55]. Therefore, *ITGAV* may contribute to some extent to chondrodystrophy in dogs with SHM. Besides this, some integrins act as receptors for several viruses [56] and *ITGAV* is implicated in virus multiplication [57].

One gene related to calcium homeostasis has been found to also be associated with the development of SHM, *HPCAL1.* Calcium acts as a messenger molecule in many tissues and organs regulating a wide variety of important cellular processes. Recently, Marchant et al. [30] identified a LINE-1 insertion variant in a gene also related to calcium homeostasis, *SMOC2,* as the underlying mutation for “face length variation”.

Finally, two other genes were found associated with SHM in FB: *E2F6,* with a crucial role in cell cycle control that has been suggested to act as an inhibitory regulator of IL-3 and allergy [58]; and *SERGEF,* encoding a secretion-regulating guanine nucleotide exchange factor of unknown function and displaying a possible pathogenic variant removing an intronic branch-site sequence (C/A, CFA21:40329686, Canfam 3.1 assembly). However, the link between both candidate genes and this disorder is still poorly defined. 

## 5. Conclusions

Seven candidate genes were identified as associated with canine SHM in dogs. Of these, four genes were determined to be related to bone development (*PLXNA2* with prognathism, *HHAT* with the complex Nivelon-Nivelon-Mabille Syndrome, *MBOAT2* with chondrocyte differentiation, and *ITGAV* with chondrodystrophy), and one to calcium homeostasis (*HPCAL1*). Among them, it is worth highlighting the association of a possible pathogenic variant generating a new intronic branch-site sequence (T/C, CFA7:7043294, Canfam 3.1 assembly) in *PLXNA2*, although further validation is needed at transcript level. Taking into account previous studies in dogs that related SHM to CCJ problems, these genes may be considered good candidates for the development of this disease. Although this report dissected the genomic component of SHM in dogs, paving the way for future research on this complex disease in both its canine and human manifestations, its limitations related to design and sample size require further studies expanding the number of samples (both SHM cases and controls), as well as reducing the variability within the control group, to validate the results obtained. 

## Figures and Tables

**Table 1:** Description of the French Bulldog individuals from the Veterinary Neurology Reference Center (VNRC) included in the study.

Group	Dog	Sex	Age (Years)	Weight (kg)	Location of SHM	Neurological Dysfunction ^a^	Intervertebral Disc Disease	Spinal Malformation ^b^	Spondyloarthrosis	Another Disease
SHM cases	1-40353	F	10.6	12.2	Cervical, thoracic, lumbar	Grade II	C2-C3, T11-T12, T12-T13, T13-L1	VM T3, T4, T7, T11	No	Brachycephalic syndrome
	2-40354	M	3.2	12	Cervical	Grade I	C3-C4	VM T5-T7	No	Brachycephalic syndrome
	3-40462	M	4.6	15.8	Cervical, thoracic, lumbar	Grade II	No	VM T5-T6	L7-S1	Stones and recurrent cystitis
	4-40468	F	7.2	11.9	Thoracic, lumbar	Asymptomatic	L6-L7, L5-L6, L7-S1	VM T4-T9	No	Heart murmur, allergies
	5-38819	F	9.9	11.8	Cervical	Grade II	C4-C5, T12-T13, L3-L4, L7-S1	VM T3-T12	Generalized	Pyometra, mammary nodules, allergies
	6-38818	F	4.5	9	Cervical, thoracic, lumbar	Grade II	C2-C3, C3-C4, C4-C5, T13-L1, L7-S1	VM T2, T6, T8	L7-S1	No
	7-38814	M	5.4	13	Thoracic	Grade II	T11-T12, T13-L1, L1-L2, L2-L3, L4-L5, L7-S1	VM T5-T9, meningocele L7	No	Cystitis
	8-39789	M	11.3	11	Cervical, thoracic, lumbar	Grade III	C4-C5, C5-C6, T12-T13, T13-L1, L1-L2, L4-L5, L7-S1	VM T2-T10	L7-S1	Scrotal tumor
	9-38127	F	7.5	-	Cervical	Grade II	C2-C3, C3-C4, C4-C5, C5-C6, C6-C7	No	No	Allergies
	10-38811	M	10.2	10.2	Cervical	Grade III	C3-C4	No	No	Brain neoplasia (no biopsy)
	11-38212	M	4.9	10.9	Cervical	Grade I	C6-C7	No	No	No
	12-38211	F	11.9	14.8	Cervical	Grade II	No	No	Lumbar	Recurrent gastritis, neoplasia (C5 lesion with inconclusive biopsy)
Common controls	30-38213	M	5.4	16	-	-	C4-C5, C5-C6, C6-C7, L7-S1	No	No	No
	31-38215	F	9.4	11.5	-	-	T5-T6, L1-L2, L2-L3	VM T5, leptomeningeal cavitation T11	No	Dislocation of the right patella, osteoarthritis in the hips, gums nodules
	32-40466	M	7	9.7	-	-	C2-C3, C5-C6, C6-C7	VM T6-T10	T9-T10, T12-T13, L7-S1	No
	33-40357	F	5	16.2	-	-	L7-S1	VM T4-T8	No	No
	34-40469	M	7.8	12	-	-	C2-C3, C5-C6, C6-C7, L4-L5, L7-S1	VM T5-T12	T5-T7, L7-S1	No
	35-40471	F	12	9.7	-	-	C4-C5, C6-C7, L1-L2, L3-L4, L4-L5, L7-S1	No	L7-S1	Biliary cholestasis, splenic neo/hyperplasia, renal cortical cysts, heart murmur
	36-40464	F	6.9	11.2	-	-	C2-C3, C3-C4, L1-L2, L2-L3	VM T5-T10	No	No
	37-39790	M	6.6	12	-	-	C2-C3, C3-C4, T12-T13, L4-L5, L7-S1	VM T3-T13	L7-S1	No
	38-38816	F	5	9.8	-	-	C2-C3, C4-C5, C5-C6, L1-L2, L7-S1	VM T3, T5, T6, T9, T13, L6, meningocele L5-L7	No	No
	39-38217	M	5	15	-	-	C3-C4, L6-L7, L7-S1	No	No	No

^a^ Neurological dysfunction grades: grade I, spinal hyperesthesia; grade II, ambulatory paresis; grade III, non-ambulatory paresis; grade IV, plegia with intact nociception; grade V, plegia without nociception. ^b^ VM Vertebral Malformation

**Table 2:** Significant associations with the development of syringohydromyelia (SHM) in dogs.

Position/Region in Canfam 3.1 Assembly	Gene Name and Symbol	Candidate Variants			
		Alleles (ref/alt)	Position	*p* Value	Gene Location/Effect
CFA7:6938927-7043294	*plexin A2 (PLXNA2)*	T/C	7043294	6.23 × 10^−11^	Intronic/Branch Site mutation
CFA7:9012453	*hedgehog acyltransferase (HHAT)*	InsT	9012453	1.80 × 10^−11^	Intronic
CFA17:6251280	*membrane bound O-acyltransferase domain containing 2 (MBOAT2)*	G/A	6251280	1.28 × 10^−9^	Upstream 5′UTR
CFA17:7242110	*hippocalcin like 1 (HPCAL1)*	C/T	7242110	1.80 × 10^−11^	Upstream 5′UTR
CFA17:8153564	*E2F transcription factor 6 (E2F6)*	A/G	8153564	1.28 × 10^−9^	Downstream 3′UTR
CFA21:40329686	*secretion regulating guanine nucleotide exchange factor (SERGEF)*	C/A	40329686	2.88 × 10^−9^	Intronic/Branch Site mutation
CFA36:28842415-28868657	*integrin subunit alpha V (ITGAV)*	A/G	28842415	6.29 × 10^−9^	Intronic

Regions are defined by variants passing the Bonferroni correction threshold (7.9) in both GWAS cohorts.

## Data Availability

Clean data is available on NCBI accession number PRJNA825150.

## Supplementary Materials

The following supporting information can be downloaded at: www.mdpi.com/article/10.3390/ani12192622/s1, Table S1: Dogs from the PRJNA448733 NCBI project (https://www.ncbi.nlm.nih.gov/bioproject/PRJNA448733) included in the analysis; Table S2: Reads statistics after removing adaptor sequences, contamination and low-quality reads; Table S3: Reads statistics after alignment to the canFam5 (UMICH_Zoey_3.1) reference genome; Figure S1: Quantile-quantile plots (QQ plots) of the genome-wide association studies (GWAS) for syringohydromyelia (SHM) development comparing (a) 12 French Bulldog (FB) SHM cases vs. 26 controls (10 FB, 16 samples from control panel 1), and (b) 12 French Bulldog SHM cases vs. 26 controls (10 FB, 16 samples from control panel 2) (Table S1). Genomes sequenced for this work are available in NCBI (Bioproject number: PRJNA825150).
