# Supplementary material for: Syringohydromyelia in Dogs: The Genomic Component Underlying a Complex Neurological Disease
Source: Animals (Basel). 2022 Sep 29;12(19):2622. doi: 10.3390/ani12192622 (PMC9558965; doi:10.3390/ani12192622)

Figure S1. Quantile-quantile plots (QQ plots) of the genome-wide association studies (GWAS) for syringohydromyelia (SHM) development comparing a) 12 French Bulldog (FB) SHM cases vs. 26 controls (10 FB, 16 samples from control panel 1), and b) 12 French Bulldog SHM cases vs. 26 controls (10 FB, 16 samples from control panel 2) (Table S1).

a)

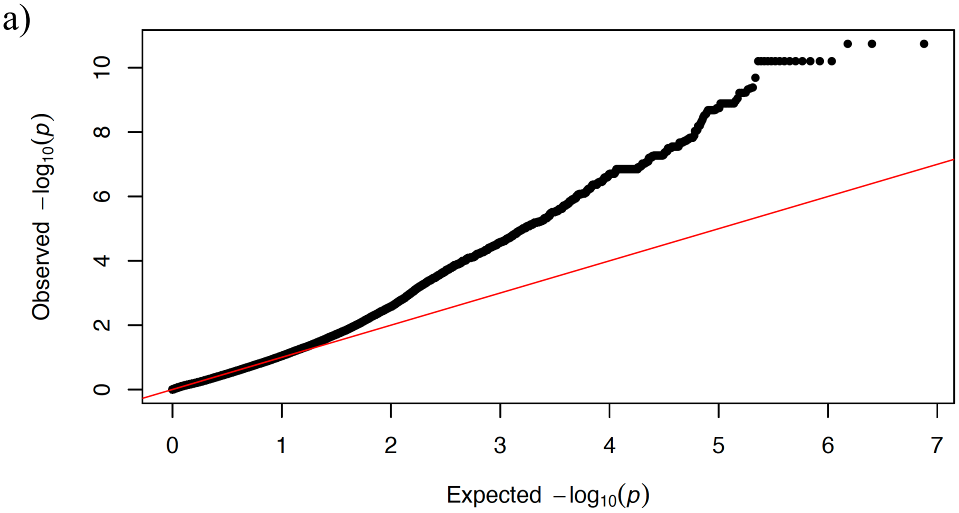

b)

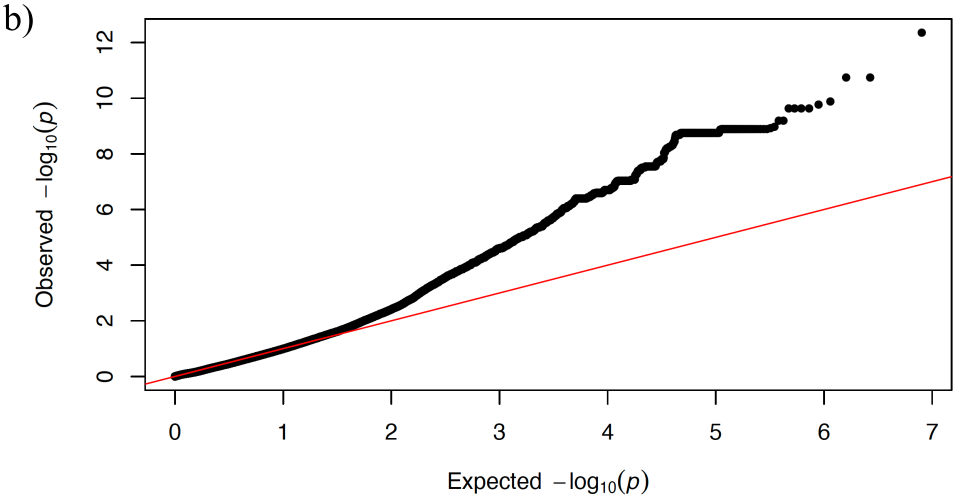

Supplement: Supplementary file 1 [file animals-12-02622-s001.zip › FigureS1.pdf]
